# Impairment in the telocyte/CD34^+^ stromal cell network in human rheumatoid arthritis synovium

**DOI:** 10.1111/jcmm.16225

**Published:** 2020-12-21

**Authors:** Irene Rosa, Maria Simonetta Faussone‐Pellegrini, Eloisa Romano, Lidia Ibba‐Manneschi, Marco Matucci‐Cerinic, Mirko Manetti

**Affiliations:** ^1^ Department of Experimental and Clinical Medicine Section of Anatomy and Histology University of Florence Florence Italy; ^2^ Department of Experimental and Clinical Medicine Division of Rheumatology University of Florence Florence Italy

**Keywords:** CD34^+^ stromal cells, human synovium, rheumatoid arthritis, telocytes

## Abstract

Telocytes (TCs)/CD34^+^ stromal cells have recently emerged as peculiar interstitial cells detectable in a variety of organs throughout the human body. TCs are typically arranged in networks establishing unique spatial relationships with neighbour cells and likely contributing to the maintenance of tissue homeostasis by both cell‐to‐cell contacts and releasing extracellular vesicles. Hence, TC defects are being increasingly reported in different pathologies, such as chronic inflammatory and fibrotic conditions. In this regard, TCs/CD34^+^ stromal cells have been shown to constitute an intricate interstitial network in the subintimal area of the normal human synovial membrane, but whether they are altered in chronic synovitis has yet to be explored. We therefore undertook a morphologic study to compare the distribution of TCs/CD34^+^ stromal cells between normal synovium and chronically inflamed synovium from patients with rheumatoid arthritis (RA) by using CD34 immunohistochemistry and CD31/CD34 double immunofluorescence. CD34 immunostaining revealed that, at variance with normal synovium, the inflamed and hyperplastic RA synovial tissue was nearly or even completely devoid of TCs/CD34^+^ stromal cells. Double immunofluorescence confirmed that almost all CD34^+^ tissue components detectable in RA synovium were blood vessels coexpressing CD31, while a widespread network of CD31^−^/CD34^+^ TCs was clearly evident in the whole sublining layer of normal synovium. In the context of the emerging diverse roles of TCs/CD34^+^ stromal cells in the regulation of tissue homeostasis and structure, the remarkable impairment in their networks herein uncovered in RA synovium may suggest important pathophysiologic implications that will be worth investigating further.

## 1 INTRODUCTION

In normal diarthrodial joints, the synovium is a thin delicate membrane attached to skeletal tissue at the bone‐articular cartilage interface that borders the joint cavities and lines tendon sheaths and bursae.^1^ It serves as an important source of joint lubricants and nutrients for articular cartilage that itself is avascular.^1^ Structurally, a healthy synovium consists of a thin (1‐3 cells thick) intimal lining layer of macrophage‐like (or type A) and fibroblast‐like (or type B) synoviocytes, and a sublining layer of loose connective tissue with blood and lymphatic vessels, nerve fibres, a few leucocytes, fibroblasts and, as only recently reported, a distinctive type of cells named telocytes (TCs)/CD34^+^ stromal cells.^1^, ^2^ Both transmission electron microscopy and light microscopy indeed revealed that TCs/CD34^+^ stromal cells, by means of their long and thin moniliform cell extensions (telopodes), shape up an intricate interstitial network extending from the lining‐sublining boundary throughout the whole subintimal area of the normal human synovial membrane.^2^ Furthermore, within the synovial sublining layer these cells appeared particularly concentrated around blood microvessels.^2^

According to numerous recent studies on different organs of the human body, TCs may establish unique spatial relationships with multiple cell types, including both neighbour parenchymal cells and other cells of the stromal compartment and are believed to act as important regulators of the homeostasis of the local microenvironment through both cell‐to‐cell contacts and releasing extracellular vesicles.^3^, ^4^, ^5^, ^6^, ^7^, ^8^ Hence, mounting evidence points towards an implication of TC defects in different pathologies, such as impairments in their stromal networks in conditions characterized by chronic inflammation and fibrotic remodelling of connective tissues.^9^, ^10^, ^11^, ^12^ In such a context, we herein considered of interest to investigate for the first time whether changes in TCs/CD34^+^ stromal cells may occur in the synovium of individuals affected by rheumatoid arthritis (RA), a disorder in which chronic synovitis leads to the formation of the so‐called synovial pannus that invades articular cartilage and bone with progressive joint destruction.^1^, ^13^

## 2 MATERIALS AND METHODS

The study was performed on archival paraffin‐embedded knee synovial tissue samples collected from four healthy individuals who underwent post‐traumatic surgical intervention (2 males and 2 females; mean [± SEM] age: 55.0 ± 2.9 years) and six age‐ and gender‐matched patients with RA who underwent surgical joint replacement (3 males and 3 females; mean [± SEM] age: 56.8 ± 2.4 years). The mean [± SEM] disease duration was 9.2 ± 1.3 years. All subjects had signed a written informed consent form. The study was carried out in accordance with the Declaration of Helsinki and approved by the Institutional Review Board at the Careggi University Hospital, Florence, Italy. Synovial sections (5 μm thick) were deparaffinized and either stained with haematoxylin and eosin for routine histopathologic analysis or subjected to antigen retrieval by boiling in sodium citrate buffer (10 mM, pH 6.0) for 10 minutes followed by immunoperoxidase‐based immunohistochemistry or double immunofluorescence staining according to previously published protocols.^2^, ^11^ Briefly, after blockade of endogenous peroxidases with 3% H_2_O_2_ solution for 15 minutes at room temperature, immunohistochemistry was carried out by incubating tissue sections overnight at 4°C with a mouse monoclonal anti‐human CD34 antibody (1:50 dilution; clone QBEnd‐10, catalog number M7165; Dako, Glostrup, Denmark) and using the UltraVision Large Volume Detection System (Anti‐Polyvalent, HRP, catalog number TP‐125‐HL; Lab Vision, Fremont, CA, USA) as described elsewhere.^2^, ^11^ Immunoreactivity was developed using 3‐amino‐9‐ethylcarbazole (AEC kit, catalog number TA‐125‐SA; Lab Vision) as chromogen followed by nuclear counterstain with haematoxylin. For immunofluorescence, tissue sections were exposed to a glycine solution (2 mg/mL) for 10 minutes to quench autofluorescence and then blocked for 1 hour at room temperature with 1% bovine serum albumin in phosphate‐buffered saline. A mixture of mouse monoclonal anti‐human CD34 (1:50 dilution; catalog number M7165; Dako) and rabbit polyclonal anti‐human CD31/platelet‐endothelial cell adhesion molecule‐1 (1:50 dilution; catalog number ab28364; Abcam, Cambridge, UK) primary antibodies was applied to tissue sections overnight at 4°C, followed by washing and incubation with Alexa Fluor‐488‐conjugated donkey anti‐mouse and Rhodamine Red‐X‐conjugated goat anti‐rabbit secondary antibodies (1:200 dilution; Invitrogen, San Diego, CA, USA) for 45 minutes at room temperature in the dark. Nuclei were counterstained with 4′,6‐diamidino‐2‐phenylindole (DAPI). Irrelevant isotype‐matched and concentration‐matched IgG were used as negative controls in all experiments. Immunostained synovial sections were observed under a Leica DM4000 B microscope and photographed with a Leica DFC310 FX 1.4‐megapixel digital colour camera equipped with the Leica software application suite LAS V3.8 (Leica Microsystems, Mannheim, Germany). CD31^−^/CD34^+^ TCs were counted in six randomly chosen microscopic high‐power fields (hpf; ×40 original magnification) per sample by two independent observers who were blinded with regard to the sample classification. Only the cells with well‐defined nuclei were counted. The mean of the two different observations for each sample was used for analysis. Statistical analysis was performed using the SPSS software for Windows Version 26.0 (SPSS, Chicago, IL, USA). Student's *t* test for independent samples was employed to analyse differences in the number of synovial TCs/hpf between control and RA groups, while Spearman's rho correlation coefficient was performed to investigate the correlation between the number of TCs/hpf in RA synovium and disease duration. *P* < .05 was considered statistically significant.

## 3 RESULTS

**FIGURE 1:**
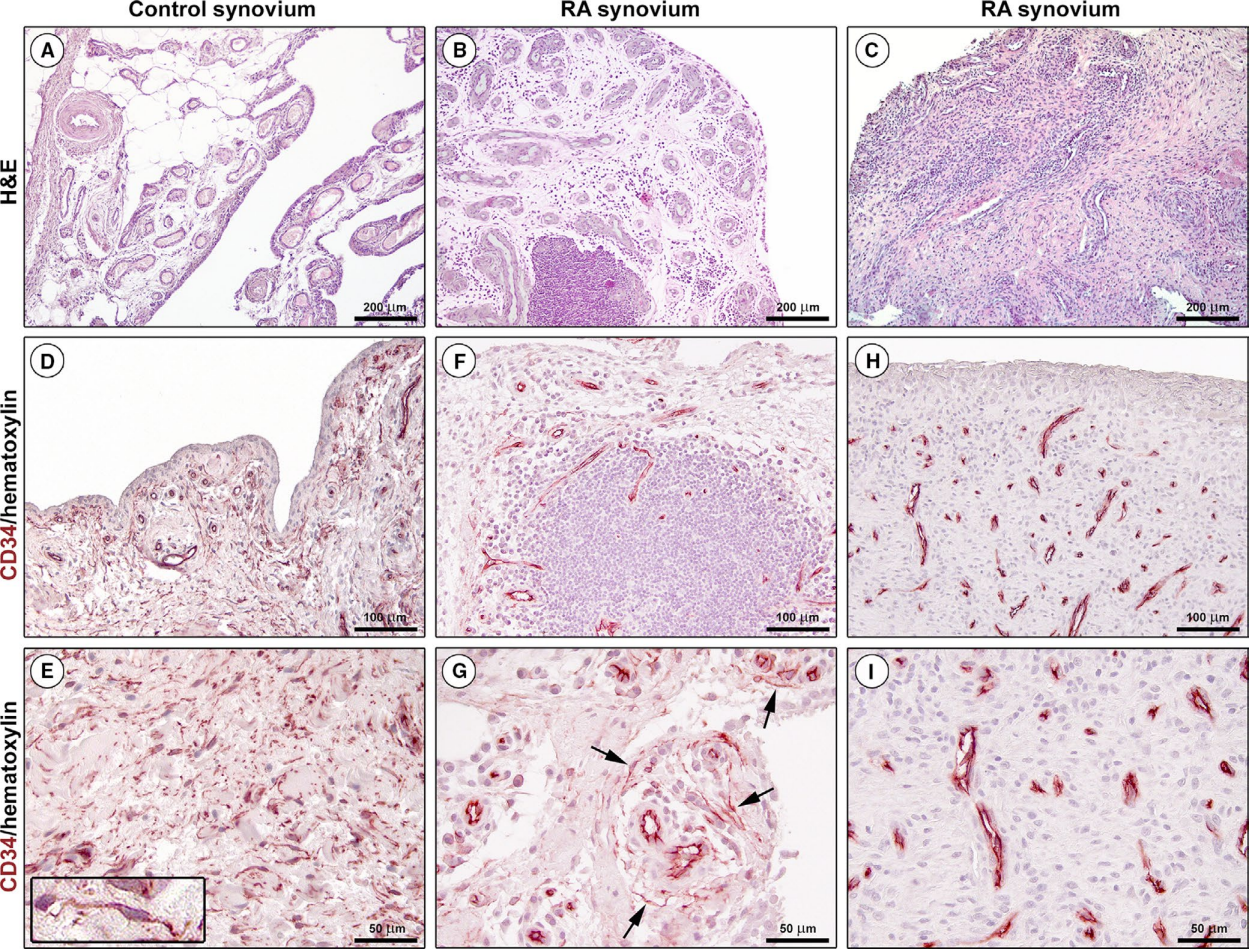
Representative light microscopy photomicrographs of synovial tissue sections from healthy controls and patients with rheumatoid arthritis (RA). (A‐C) Haematoxylin and eosin (H&E) staining. Healthy synovium consists of a thin intimal lining layer and a sublining layer of vascularized loose connective tissue with only very few leucocytes (A). Hyperplastic RA synovium displays either diffuse immune cell infiltrates or ectopic lymphoid structures in the sublining layer (B, C). (D‐I) CD34 immunohistochemistry with haematoxylin counterstain. An intricate network of telocytes (TCs)/CD34^+^ stromal cells is present in the whole sublining layer of healthy control synovium (D, E). At higher magnification, TCs/CD34^+^ stromal cells appear as spindle‐shaped cells with a small nucleated body and long cytoplasmic processes with an irregular calibre (inset in E). CD34 immunoreactivity is observed also in endothelial cells of blood microvessels. Note the presence of a few perivascular TCs/CD34^+^ stromal cells in the sublining layer of a RA synovial sample (F, arrows in G). Note the complete absence of TCs/CD34^+^ stromal cells in another RA synovial sample; all CD34^+^ tissue structures are identifiable as blood microvessels (H, I). Scale bar: 200 μm (A‐C), 100 μm (D, F, H), 50 μm (E, G, I)

As compared with healthy control synovium, the main histopathologic features of samples from RA patients were synovial hyperplasia and the presence of either diffuse immune cell infiltrates or ectopic lymphoid aggregates in the sublining layer (Figure 1A‐C). An intricate interstitial network of TCs/CD34^+^ stromal cells was distributed throughout the subintimal area of healthy control synovium (Figure 1D, E). In the synovial sublining layer, CD34 immunoreactivity was clearly evident also in the endothelium of blood microvessels (Figure 1D, E). As shown in Figure 1F‐I, the inflamed and hyperplastic RA synovial membrane was nearly or even completely devoid of TCs/CD34^+^ stromal cells. In some RA specimens, a few TCs/CD34^+^ stromal cells were observed in a perivascular location (Figure 1G), while in others blood microvessels appeared as the only CD34^+^ tissue components detectable (Figure 1I).

**FIGURE 2:**
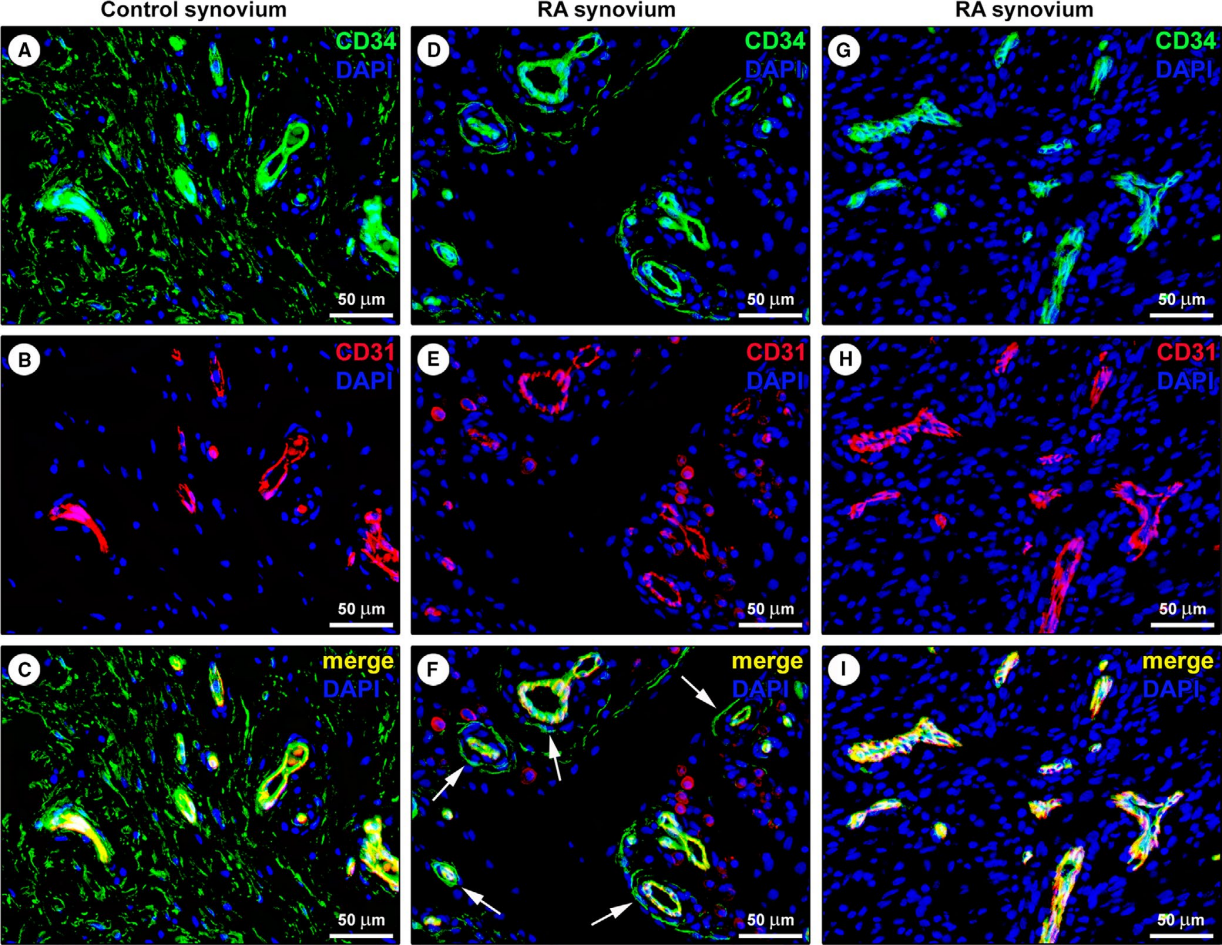
Representative fluorescence microscopy photomicrographs of synovial tissue sections from healthy controls and patients with rheumatoid arthritis (RA). (A‐I) Double immunofluorescence labelling for CD34 (green) and CD31 (red) with DAPI (blue) counterstain for nuclei. In healthy control synovium, telocytes (TCs)/CD34^+^ stromal cells lacking CD31 immunoreactivity form an extensive network in the sublining layer; endothelial cells of blood microvessels are CD31^+^/CD34^+^ (A‐C). Note the presence of a few CD31^−^/CD34^+^ TCs around CD31^+^/CD34^+^ blood microvessels in the sublining layer of a RA synovial sample (D‐F, arrows in F). In another RA synovial sample, the sublining layer displays numerous CD31^+^/CD34^+^ blood microvessels, but CD31^−^/CD34^+^ TCs are undetectable (G‐I). Scale bar: 50 μm (A‐I)

Since CD34 immunoreactivity was also displayed by endothelial cells that may be misidentified as TCs when blood microvessels are sectioned tangentially with no obvious lumen, we further carried out CD31/CD34 double immunofluorescence to distinguish with certainty between CD31^−^/CD34^+^ TCs and CD31^+^/CD34^+^ vascular structures (Figure 2). This analysis confirmed the presence of a widespread network of CD31^−^/CD34^+^ TCs surrounding CD31^+^/CD34^+^ blood microvessels and extending in the whole sublining layer of healthy control synovium (Figure 2A‐C). In RA synovium, CD34 immunoreactivity was observed almost exclusively in numerous CD31^+^/CD34^+^ blood microvessels, except for a small number of perivascular CD31^−^/CD34^+^ TCs found in some samples (Figure 2D‐I). Quantitative analysis revealed that the number of CD31^−^/CD34^+^ TCs/hpf was significantly reduced in RA compared with healthy control synovial specimens (mean [± SEM]: 3.6 ± 0.8 *versus* 21.8 ± 1.2, *P* < .0001). Of note, the number of CD31^−^/CD34^+^ TCs/hpf in RA synovium resulted inversely correlated with disease duration (Spearman's rho = −0.83, *P* = .04).

## 4 DISCUSSION

It is widely acknowledged that characteristic histopathologic features of RA synovitis include hyperplasia of the intimal lining layer due to an accumulation of macrophages and an uncontrolled proliferation of activated fibroblast‐like synoviocytes, as well as sustained neoangiogenic processes and a vast influx of immune cells of both the innate and adaptive immune system in the synovial sublining.^1^, ^13^ Our histologic data add to this scenario highlighting that RA synovitis is also characterized by a remarkable decrease in CD34 staining due to a severe impairment in the network of TCs/CD34^+^ stromal cells throughout the whole synovial subintimal area.

Of note, these findings are in line with those of a number of studies that reported a reduction/loss of TCs/CD34^+^ stromal cells in a variety of chronically inflamed tissues in different diseases.^9^, ^10^, ^11^, ^12^ Considering the emerging diverse roles proposed for the TCs/CD34^+^ stromal cells,^4^, ^5^, ^9^ we are confident that our preliminary results lay the groundwork for future research aimed at clarifying how the changes described here might contribute to alter the synovial microenvironment in RA. Indeed, among the functions attributed to these cells is noteworthy their possible involvement in the regulation of local tissue immune homeostasis and angiogenesis, which are profoundly disturbed in RA synovium.^1^, ^2^, ^13^, ^14^ In RA, chronic synovitis is thought to be maintained mainly by complex interactions of cell types native to the joint and inflammatory cells recruited into the joint through a plethora of cytokines,^1^, ^14^ and the impairment in the TC/CD34^+^ stromal cell network might be implicated in such pathogenic mechanisms. Moreover, it is well recognized that stromal cells are leading actors in shaping the organization, integrity and dynamics of their own microenvironment, but their phenotype and functions also are tightly dependent on the specific tissue microenvironment where they reside. Therefore, it will be interesting to further investigate in depth whether during RA synovitis these cells simply degenerate and disappear or may change their immunophenotype by losing CD34 expression. In this regard, it is worth mentioning that a previous work noticed an expansion of a fibroblast population expressing podoplanin, CD90/Thy1 and cadherin‐11, but lacking CD34, in patients with RA relative to patients with osteoarthritis.^15^ Electron microscopy analyses could help in identifying the presence of degenerative changes in TCs within the RA synovium. Since initial changes in TCs/CD34^+^ stromal cells cannot be studied in chronically affected synovium, a further study of these cells in synovial tissues with early inflammatory processes would be appropriate to ascertain whether they may behave as precursor cells for myofibroblasts arising in granulation tissue, with loss of CD34 and parallel acquisition of α‐smooth muscle actin expression.^16^ Whether similar alterations in the network of TCs/CD34^+^ stromal cells may be part of synovial histopathology in arthritic diseases other than RA should also be verified. Hopefully, future studies will explore these exciting possibilities.

## CONFLICT OF INTEREST

The authors confirm that there are no conflicts of interest.

## AUTHOR CONTRIBUTIONS

**Irene Rosa:** Data curation (equal); Formal analysis (equal); Investigation (equal); Methodology (lead); Writing‐original draft (equal); Writing‐review & editing (equal). **Maria‐Simonetta Faussone‐Pellegrini:** Data curation (equal); Formal analysis (equal); Investigation (equal); Supervision (equal); Writing‐original draft (equal); Writing‐review & editing (equal). **Eloisa Romano:** Formal analysis (equal); Investigation (equal); Methodology (equal); Writing‐review & editing (equal). **Lidia ibba:** Data curation (equal); Formal analysis (equal); Investigation (equal); Writing‐review & editing (equal). **Marco Matucci‐Cerinic:** Investigation (equal); Resources (equal); Supervision (equal); Writing‐review & editing (equal). **Mirko Manetti:** Conceptualization (lead); Data curation (equal); Formal analysis (equal); Funding acquisition (lead); Investigation (equal); Methodology (equal); Resources (equal); Supervision (lead); Writing‐original draft (lead); Writing‐review & editing (equal).

## Data Availability

The data that support the findings of this study are available from the corresponding author upon reasonable request.
